# Malignancy Risk and Hormonal Activity of Adrenal Incidentalomas in a Large Cohort of Patients from a Single Tertiary Reference Center

**DOI:** 10.3390/ijerph16101872

**Published:** 2019-05-27

**Authors:** Ewa Cyranska-Chyrek, Ewelina Szczepanek-Parulska, Michal Olejarz, Marek Ruchala

**Affiliations:** Department of Endocrinology, Metabolism and Internal Medicine, Poznan University of Medical Sciences, 60-355 Poznań, Poland; ewelinaparulska@gmail.com (E.S.-P.); ml.olejarz@gmail.com (M.O.); mruchala@ump.edu.pl (M.R.)

**Keywords:** adrenal tumor, adrenal incidentaloma, adrenal cancer, pheochromocytoma, primary hyperaldosteronism, autonomous cortisol secretion

## Abstract

Background: A rise in adrenal incidentalomas (AIs) detection has been observed recently. Even though AIs are detected in asymptomatic patients, thorough assessment may reveal hormonal and metabolic abnormalities or malignant character. Methods: Medical records of 2005 patients (1301 women, 704 men) with 2498 tumors aged 61 ± 11.3 (18–93) years, who had been hospitalized due to AI diagnosis, were reviewed. Patients underwent clinical examination, adrenal CT and hormonal assessment. In patients subjected to adrenalectomy, histopathological character of AI was confirmed. Results: AIs most frequently occurred in patients in their 7th decade of life. Hypertension was present in 76.6%, glucose metabolism disorders in 41.3%, and hypercholesterolemia in 60.1% of patients. Lipid-rich adenomas (83.2%) and hormonally inactive tumors (83.1%) predominated. Autonomous cortisol secretion was present or suspected in 6.6%, pheochromocytoma in 4.7%, hyperandrogenism in 3.1%, and primary hyperaldosteronism in 2.4% of patients. The risk of malignancy increased in patients with tumors >6 cm was 37.7%. The logistic regression analysis revealed that the strongest predictor of hormonal activity of AIs was lipid-poor picture on CT scan (OR 7.072; CI 5.118–9.771), while the most important factor increasing the risk of malignancy was lipid-poor adenoma or non-adenoma on CT scan (OR 4.843; CI 1.697–13.819). Final histopathology was available for 214 tumors; 106 adrenocortical adenomas, 46 pheochromocytomas, and 18 adrenocortical carcinomas were diagnosed. Conclusion: Most AIs are hormonally inactive adenomas. The most frequent hormonal manifestation of AI is subclinical hypercortisolemia. Presence of AI is often accompanied by features of metabolic syndrome. The tumor density on CT scan picture may be predictive of both hormonal activity and the risk of malignancy. Tumors of all sizes may exhibit hormonal activity, while the risk of malignancy significantly increases with the size above 6 cm.

## 1. Introduction

In the last decades we have witnessed an epidemic increase in the frequency of adrenal incidentalomas (AIs) diagnosis. The first AI was described in 1982 [1]. Dynamic development of imaging techniques, such as ultrasonography (US), CT or MRI, together with their constantly increasing availability, resulted in a rapid rise in new AI diagnoses. The imaging studies of the thorax, aiming at early detection of lung cancer among tobacco smokers, are another contributing factor. In 1994, Griffing accurately referred to this phenomenon as “a disease of modern technology” and “new epidemy of A-I-D-S (adrenal incidentaloma discovered serendipitously)” [2]. Therefore, AIs constitute an important clinical issue, which may constitute a diagnostic and therapeutic dilemma, posing significant burden for healthcare worldwide.

An AI is defined as any adrenal focal lesion found incidentally during imaging examinations (US, CT, MRI), performed due to extra-adrenal causes [3], whose tumor size is 1 cm or more in diameter [4]. Adrenal tumors are detected in about 0.3–1.3% of CT examinations. However, some authors estimate the percentage to be 4% in oncological patients and even 10–15% during autopsies. Adrenals constitute the fourth most frequent metastatic site for malignant cancers, especially breast, prostate, colon and renal cancer, as well as skin melanoma. The frequency of appearance correlates with patients’ age; adrenal masses are rare under the age of 30, while are more frequently diagnosed over 70 years of age [5]. Adrenal tumors are detected 2.5-fold more often in women. However, metastases to adrenal glands are the only exception and dominate in men. In 9–22% of cases, adrenal lesions are bilateral [6]. The largest multi-center analysis of a group of patients with AIs so far was conducted in Italy and included 1013 patients with AI, 786 of which had full hormonal assessment [7]. However, the recent literature lacks comprehensive evaluation of a large cohort of patients with incidentally detected adrenal masses.

Thus, the aim of the study was to assess the malignancy rate and hormonal activity of AIs detected in patients hospitalized in a single tertiary reference endocrine center.

## 2. Materials and Methods

This retrospective and descriptive cross-sectional study was approved by the local Bioethical Committee (approval number 397/12). Medical records of patients hospitalized in the period from January 1996 to December 2015 in a single tertiary reference endocrine center were reviewed to identify patients admitted due to diagnosis of AI. Patients referred to the department due to any clinical or biochemical suspicion of hormonally active adrenal tumor or adrenal cancer were excluded from the study. Patients for whom imaging was performed for renal artery visualization due to an isolated arterial hypertension, or diagnostics during oncological follow-up due to malignant neoplasms different than adrenal cancer, were also included. Finally, 2005 consecutive patients (1301 women, 704 men) with 2498 AIs met the inclusion criteria.

Descriptive data will be presented as mean ± SD, while categorical data as number and percentage (%). All patients were evaluated to assess the morphology, the risk of malignancy, and hormonal activity of the tumor. Patients underwent full clinical examination, CT focused on adrenal glands, and biochemical tests. The following parameters were analyzed: serum cortisol circadian rhythm, morning ACTH concentration, cortisol following 1 mg dexamethasone suppression test, DHEA-S concentration. Moreover, urine was collected to assess the 24 h excretion of cortisol, normetanephrine and metanephrine. In addition, in patients with hypertension or hypokalemia, aldosterone concentration and plasma renin activity in tilt test were measured and aldosterone-renin ratio (ARR) was calculated. The type of adrenal lesion was classified based on a detailed description of density in Hounsfield Units (HU) and the profile of contrast medium washout was determined by experienced radiologists. Lipid-rich adenomas were characterized as such if they were low density on CT scan (<10 HU). In these cases there is no need to perform contrast medium washout evaluation. Lipid-poor adenomas were defined as lesions if characterized by the density on CT scan was between 10–20 HU and the contrast media washout was >60%. Non-adenomas were defined as lesions characterized by density on CT scan above 20 HU and contrast media washout <60%. The types and criteria of hormonal secretion of adrenal tumors adopted to characterize our cohort were based on modified and combined recommendations provided in the current guidelines [8,9], and are presented in the Table 1.

Patients without contraindications for surgery, in whom a malignancy or hormonal activity of AI was suspected, or the size of the tumor exceeded 4 cm, were consulted surgically, and adrenalectomy was considered.

The data were subjected to statistical analysis with the use of Statistica 12 (StatSoft, Krakow, Poland) software. The normality of the distribution of variables was verified by the Shapiro-Wilk test. The Pearson’s linear correlation coefficient (r) was used to determine the relationship between individual variables. Logistic regression analysis was performed to find the determinants of hormonal activity or malignant character of AIs. Statistical significance was defined as a p value less than 0.05.

## 3. Results

**Figure 1:**
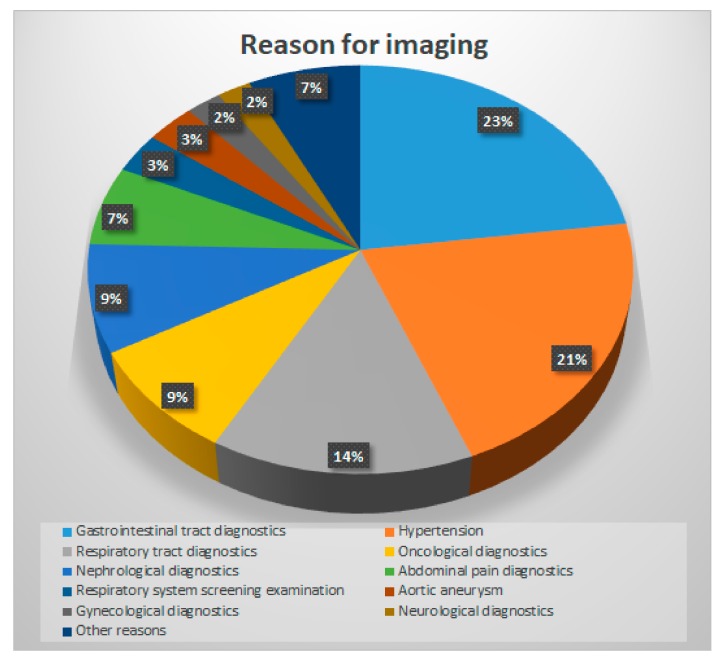
Causes for the first imaging examination, which revealed the presence of adrenal incidentaloma.

The reasons to perform diagnostic imaging in our cohort of patients are presented in the Figure 1.

Mean age of patients was 61 ± 11.3 (18–93 years). Adrenal incidentalomas more often occurred in patients of older age: 526 (26.2%) of patients were diagnosed in patients in the 6th decade of life (aged 50–59 years), 797 (39.8%) of patients were diagnosed in their 7th decade of life (aged 60–69 years), and 346 (17.3%) were diagnosed in the 8th decade of life (aged 70–79 years). The mean BMI of patients with AIs was 28.8 ± 5.1 kg/m^2^; 752 patients (37.5%) were overweight, whereas 767 patients (38.3%) were obese. The majority of patients (1535 subjects, 76.6%) were diagnosed with hypertension, while 828 patients (41.3%) suffered from glucose metabolism disorders: impaired glucose tolerance (IGT) and/or increased fasting glucose (IFG) in 301 cases (15%), diabetes in 527 patients (26.3%). The majority of patients presented with dyslipidemia: hypercholesterolemia (defined as total cholesterol TChol ≥ 200 mg/dL) was found in 1206 patients (60.1%), increased LDL cholesterol ≥ 135 mg/dL in 792 (39.5%) of patients, and hypertriglyceridemia (triglycerides TG ≥ 150 mg/dL) in 646 (32.2%) of patients.

Unilateral lesions were predominant (demonstrated in 1552 patients which accounts for 77.4% of AIs). Most AIs were diagnosed by means of CT scan. Lipid-rich adenomas were predominant (2044 tumors, 81.8%), while lipid-poor adenomas (327; 13.1%) and non-adenomas (92; 3.7%) were less frequent.

**Figure 2:**
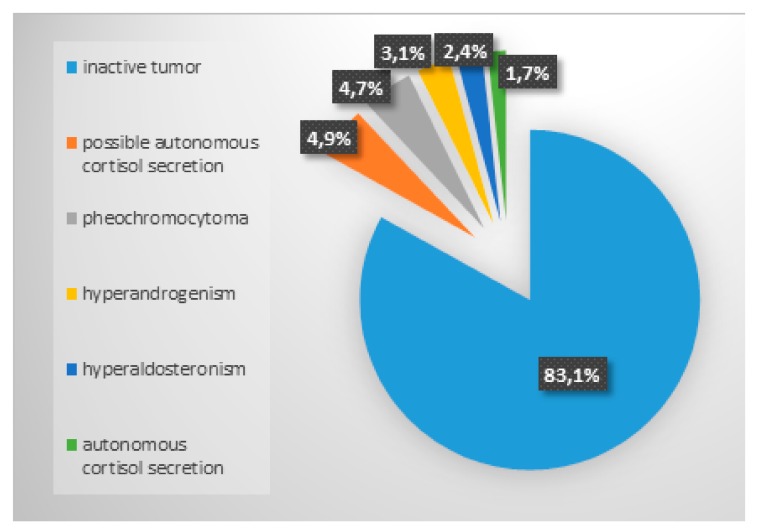
The results of hormonal activity assessment of adrenal incidentalomas.

In the course of endocrine diagnostics, the majority of tumors turned out to be hormonally inactive (1666 (83.1%) of patients). The most common disorder was possible autonomous cortisol secretion. The distribution of particular types of hormonal activity is presented on the Figure 2. In 2248 (90%) of tumors the maximum size of AI did not exceed 4 cm.

There was a positive correlation between manifestation of hormonal activity and increasing tumor size. Among patients with tumors <4 cm, hormonal activity was detected in 14.6% of patients, in patients with tumor size between 4–6 cm, in 37% of patients, while in patients with tumors >6 cm in 51.2% of patients.

**Figure 3:**
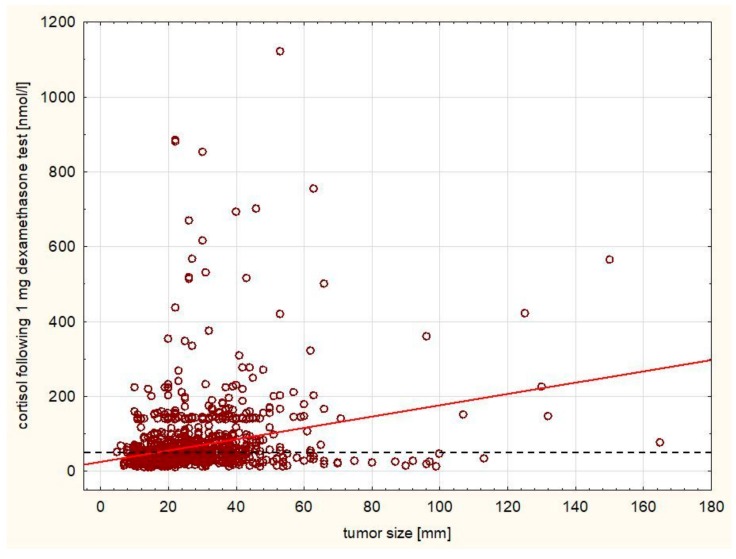
Positive correlation between the size of the tumor and morning cortisol level following 1 mg dexamethasone test (r = 0.2628, *p* < 0.005). The cut-off value for normal range is marked with a dashed line.

**Figure 5:**
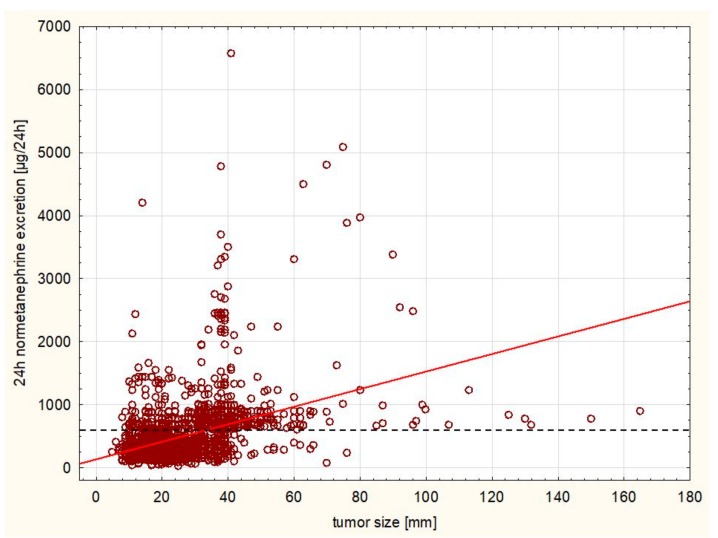
Positive correlation between the size of the tumor and 24 h normetanephrine excretion (r = 0.4004, *p* < 0.005). The cut-off value for normal range is marked with a dashed line.

A statistically significant positive correlation was observed between the size of the tumor and morning cortisol level following 1 mg dexamethasone test (r = 0.2628, Figure 3) as well as the 24 h cortisol excretion and size (r = 0.1542, Figure 4), while there was a negative correlation between ACTH level and size (r = −0.0411). There was a significant negative correlation between aldosterone level at rest and size of the tumor (r = −0.1268). A significant positive correlation was observed between the size of the tumor and 24 h metanephrine (r = 0.2866) and normetanephrine excretion (r = 0.4004, Figure 5) urine excretion. A positive correlation was observed also between DHEA-S concentration and size of the tumor (r = 0.0624).

The risk of malignancy increased with the tumor size. In patients with tumors <4 cm, the malignancy rate was 0.2%, and 4.8% in the 4–6 cm group, while the rate in patients with tumors >6 cm was 37.7%.

The logistic regression analysis revealed that hormonal activity could be predicted by the presence of lipid-poor adenoma picture on CT scan (OR 7.072; CI 5.118–9.771). In 20% of patients, surgical procedure was considered due to tumor hormonal activity and/or the size of the adrenal lesion >4 cm. Final histopathological diagnoses of the tumors were available for 214 patients and are demonstrated in the Table 2. A separate analysis was performed for patients who eventually underwent adrenalectomy. The most important predictor of malignant character of the lesion was the CT scan picture of lipid-poor adenoma or non-adenoma (OR 4.843; CI 1.697–13.819).

## 4. Discussion

To the best of our knowledge, our study represents one of the largest cohorts of patients with AIs reported carried out to date. However, retrospective character of the study and inclusion of patients for whom imaging was performed during oncological follow-up or with an isolated arterial hypertension might constitute potential limitations of our analysis and reason for bias.

Our findings are in agreement with previous observations, that prevalence of AIs tends to increase with age [10,11,12]. Thus, due to aging of societies and longer lifespans, the detection rate in case of AIs is predicted to rise. In fact, the AI detectability between 40–49 years of age is estimated at 3–5%, increasing to 8–10% in the 8th decade of life. However, in our cohort, the majority of AIs were detected in the 7th decade of life. Moreover, similar to our cohort, the majority of research indicates that women are more likely to develop AI (women to men ratio is 3:1).

In our study 83.1% of tumors were hormonally inactive. In previous analyses, the percentage of hormonally inactive tumors was 70–94% [13]. In the largest so far carried out with an Italian group of patients with AIs, 89% of tumors turned out to be hormonally non-functional [7]. Interestingly, many of patients with AIs present features of metabolic syndrome. Thus, the question remains whether it is a result of subclinical hypercortisolemia or the result of the fact that the comorbidities of these patients are more thoroughly diagnosed. There is growing evidence suggesting that the presence of AI is often associated with the presence of factors increasing cardio-metabolic risk, such as hypertension, insulin resistance, hyperglycemic states and dyslipidemia [7,13,14]. Similar to our observations, in a cohort of patients described by Ribeiro Cavalari et al., metabolic syndrome was significantly more frequent in patients with non-functional AIs than in control subjects with no adrenal tumor (69.2% vs. 31.0% accordingly) [15]. This may suggest a bidirectional relationship between the presence of AIs and insulin resistance, potentially promoting growth of adrenal masses, but also subclinical hormonal activity of AIs leading to metabolic disturbances.

The overnight 1 mg dexamethasone suppression test is a well-recognized procedure to exclude autonomous cortisol secretion according to the latest guidelines [9]. The cut-off point is often reported to be equal to <1.8 mcg/dL (<50 nmol/L). Frequently, the interpretation of the test result is limited by parameters affecting the reliability of the test and thus the possibility of a false negative result of the suppression test. One of the most common reasons of false negative results of this test are CYP3A4 cytochrome inhibitors (which is a component of P-450 cytochrome), which include commonly prescribed antidepressants and antipsychotics, antibiotics, azole antifungal drugs and other drugs or food components. Thus, in our cohort we have chosen not to interpret this test alone, but in the context of ACTH and cortisol diurnal rhythm and urine excretion for cortisol.

The analysis of our cohort of patients indicates that hormonal secretion of the tumor is weakly correlated to the tumor size. Although these correlations are statistically significant (presumably due to very large sample size), hormonal oversecretion occurs even in smaller tumors. Thus, it appears that an evaluation of hormonal secretion of the adrenal tumors should be evaluated for all tumor sizes.

The radiological diagnosis of pheochromocytoma remains a diagnostic challenge. Such tumors are characterized by phenotypic imaging variety, as they can imitate any type of tumor from adenoma, non-adenoma to cancer and metastatic lesions [16]. Blake et al. report that pheochromocytoma may present as an adenoma of density <10 HU and be characterized by high washout coefficient (>60%); thus, this may fulfill criteria for lipid-rich adenoma [17]. Kopetschke et al. assess that up to 30% of pheochromocytomas are detected incidentally, whereas the typical clinical symptoms (triad: diaphoresis, headache, palpitations) are present only in about 10% of patients [18]. In the study by Montero et al., 3.4% of AIs were eventually diagnosed as pheochromocytoma [7]. Other studies report the incidence of pheochromocytoma at the level of 1.5–14% [9]. In our cohort, 4.7% of patients were suspected of the presence of pheochromocytoma. Out of the patients referred for surgery, 21.5% turned out to be pheochromocytoma, compared to 11–23% reported in the literature [9]. The variability of the presentation of pheochromocytoma suggests diagnostics towards pheochromocytoma for all AI cases. Moreover, recent studies indicate that up to 25% of pheochromocytoma patients have a genetic background (NF1, VHL, SDHD, SDHB or RET gene mutations) and thus both the patients and their closest blood relatives require wide multidisciplinary diagnostics as well as long-term follow-up [18,19].

The prevalence of aldosteronoma in the AI population is estimated at 1.4–5% depending on the study [20,21], compared to 2.4% in our study. Until recently, the coincidence of adrenal tumor, resistant hypertension and spontaneous hypokalemia clearly implied primary hyperaldosteronism. However, novel research indicates a more frequent prevalence of primary hyperaldosteronism for patients also suffering from mild to moderate hypertension and normokalaemia [22]. In fact, patients with excess secretion of aldosterone present normokalaemia in 7–38% of cases [23,24,25], including patients with resistant hypertension [26]. Unilateral adrenal adenoma is a cause of aldosterone excess in 30–60% of patients with primary hyperaldosteronism (PA) [27]. However, one must bear in mind that the most frequent cause of PA is bilateral adrenal hyperplasia.

Moreover, there is an important limitation of routine biochemical diagnostics and imaging of AI towards primary hyperaldosteronism. There is a dangerous possibility of imaging a non-functioning adenoma, whereas in fact the hormone excess is produced by the contralateral microadenoma, which was not demonstrated by CT. Thus, in these patients, lateralization of aldosterone secretion ought to be further verified by adrenal venous sampling. Owing to this method, PA can turn out to be caused by a pathology localized on the opposite side to the tumor detected in the adenoma imaging examination [28,29].

Adrenocortical carcinomas constitute as many as 5% of AI cases. In our cohort, this type of cancer was finally diagnosed in 8.4% of subjects referred for adrenalectomy. They are characterized by poor prognosis as the 5-year survival is estimated at 20–35% [30]. In addition, they can be asymptomatic for a long time and be hormonally silent, as they show hormonal activity only in about 4–15% of cases [31,32]. The most common clinical manifestation in the hormonally active carcinomas is hypercortisolemia and/or virilization [33]. New schemes have emerged in AI malignancy risk assessment, due to lack of or a delayed detection of carcinomas. Recent recommendations of the National Institute for Health and American Association for Clinical Endocrinologists estimated the risk of carcinogenesis at 25% for tumors with the diameter >6 cm, 5% for tumors of maximum size between 4–6 cm, and 2% for the diameter <4 cm [34]. Sturgeon et al. demonstrated that the risk of carcinogenesis in tumors with the diameter ≥4 cm is doubled and reaches 10%. They suggest qualification on the basis of additional features, i.e., recommend adrenalectomy in the case of tumors >3 cm in diameter in young patients with low perioperative risk, whereas for older patients with concomitant diseases, the tumor qualification is diameter >5 cm [35]. Other authors recommend adrenalectomy with tumor size of 3 cm [36], 4 cm [37,38], 5 cm [39], 6 cm [40]. Birsen et al. recently suggested a new stratification scheme for the AI patients’ risk. It is based on points added for the size of the tumor (1, 2, 3 points for the diameter of 4 cm, 4–6 cm, and 6 cm respectively), and its density in CT (1, 2, 3 points for the density ≤10 HU, 10–20 HU, ≥20 HU). The authors emphasize the necessity of surgical excision of any hormonally active adrenal lesion, surgical removal of hormonally inactive lesions which received 2–4 points only in case of blurred margins or heterogeneity, and all lesions with 5–6 points [41]. In our study the risk of malignancy increased with the tumor size; in patients with tumors <6 cm, the malignancy rate was <5%, however in patients with tumors >6 cm increased to 37.7%.

## 5. Conclusions

Despite the fact that most patients with AIs present with lipid-rich hormonally inactive adenomas with the diameter <4 cm and do not require surgical intervention, about in one fifth of AIs, surgical treatment should be considered due to hormonal overactivity and/or malignancy risk. In hormonally active AIs, the most frequent hormonal manifestation is subclinical hypercortisolemia. Presence of AI is often associated with coexisting features of the metabolic syndrome. The tumor density on CT scan picture may be predictive of both hormonal activity and the risk of malignancy. Tumors of all sizes may exhibit hormonal activity, while the risk of malignancy significantly increases with the size above 6 cm.

## Figures and Tables

**Figure 4:**
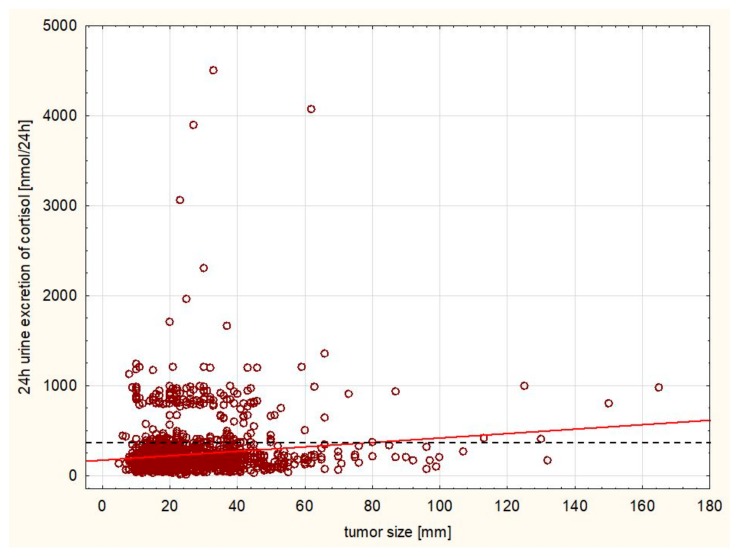
Positive correlation between the 24 h cortisol excretion and size (r = 0.1542, *p* < 0.005). The cut-off value for normal range is marked with a dashed line.

**Table 1:** The established diagnostic criteria of hormonal activity of adrenal tumors.

Diagnosed Type of Hormonal Activity	Diagnostic Criteria
**Possible autonomous cortisol secretion**	Diagnosed if at least three out of four mentioned features were present: (1) ACTH suppression < 7.20 pg/mL (2) disturbed circadian rhythm of cortisol secretion (lack of decrease in cortisol level by at least 50% at 6 p.m. in comparison to 8 a.m.) (3) increased 24 h urinary cortisol excretion (by at least two times above upper normal limit) (4) lack of cortisol suppression during 1 mg dexamethasone test (cortisol level at 8 a.m. >50 nmol/L)
**Autonomous cortisol secretion**	Diagnosed if all four features mentioned above were present
**Pheochromocytoma**	Diagnosed if 24 h urinary excretion of catecholamines metabolites (normetanephrine and/or metanephrine) was significantly increased (by at least two times above upper normal limit)
**Primary hyperaldosteronism**	Diagnosed when all features below were present: (1) increased concentration of aldosterone at rest (2) lack of significant decrease of aldosterone level during tilt test (3) plasma renin activity below normal range (4) Aldosterone-to-renin ratio (ARR) > 20
**Adrenal hyperandrogenism**	Increased DHEA-S concentration (above normal range defined according to age)

**Table 2:** Histopathological diagnosis of adrenal incidentalomas following adrenalectomy.

Histopathological Diagnosis	Number (%) of Patients
Adrenocortical adenoma	106 (49.5%)
Pheochromocytoma	46 (21.5%)
Adrenocortical carcinoma	18 (8.4%)
Renal cell cancer	10 (4.7%)
Follicular hyperplasia	6 (2.8%)
Neurofibroma (3), Hamartoma (3)	6 (2.8%)
Calcified cyst	5 (2.4%)
Myelolipoma	5 (2.4%)
Metastasis from Lung Cancer (2), Metastasis from Breast Cancer (2)	4 (1.9%)
Oncocytoma	2 (0.9%)
Angiomyolipoma	2 (0.9%)
Ganglioneuroma	2 (0.9%)
Choristoma (1), Liposarcoma (1)	2 (0.9%)
**Total**	**214**
